# Multiple eruptive dermatofibromas occurred after receiving sequential treatment with secukinumab, guselkumab, and adalimumab: case report

**DOI:** 10.3389/fimmu.2025.1611831

**Published:** 2025-11-07

**Authors:** Weiquan Chen, Linglu Fang, Ying Zhou

**Affiliations:** 1 Institute of Dermatology and Venereal Diseases, Affiliated Hospital of Guangdong Medical University, Zhanjiang, Guangdong, China; 2 Department of Dermatology and Venereal Diseases, Affiliated Hospital of Guangdong Medical University, Zhanjiang, Guangdong, China

**Keywords:** dermatofibromas, psoriasis, biological therapies, case report, guselkumab, secukinumab, adalimumab

## Abstract

Multiple eruptive dermatofibromas (MEDFs) are characterized by the rapid development of multiple dermatofibromas within a short period, often associated with underlying immune dysregulation or immunosuppressive therapies. We report a rare case of MEDFs in a young male with refractory psoriasis, who developed multiple cutaneous tumors following sequential treatment with biologic agents: secukinumab, guselkumab, and adalimumab. Despite achieving partial control of psoriasis, the patient experienced the onset of widespread, asymptomatic dermatofibromas, leading to the cessation of biologic therapy. Clinical examination, dermoscopic evaluation, and histopathological analysis confirmed the diagnosis of MEDFs. The Naranjo algorithm and WHO-UMC scale suggested a probable adverse drug reaction as the causative factor. The pathogenesis may involve a Th2-polarizing immune shift and persistent activation of antigen-presenting cells, possibly triggered by the cumulative effects of the biologics used. However, as a single-case report, our findings require validation through larger cohort studies to establish causality and incidence. This case highlights the potential for MEDFs as a novel adverse effect of biologic therapies and underscores the need for awareness and monitoring of such reactions in clinical practice.

## Introduction

Dermatofibroma (DF) is a benign, asymptomatic dermal tumor of fibrohistiocytic origin. Multiple eruptive dermatofibromas (MEDFs) are defined as a rare variant characterized by the rapid development of at least 15 lesions within one year or 5 to 8 lesions developing over a 4-month period (1) MEDFs herald an underlying immune alteration, and up to 80.1% of MEDFs patients have immune-mediated disorders or medication, or both, primarily systemic lupus erythematosus, HIV, and hematologic malignancies (2). Other associations also reported include pregnancy, Down syndrome, hypertriglyceridemia, myasthenia gravis, pemphigus vulgaris, immunosuppressants (corticosteroids and cyclophosphamide), biologics, and antineoplastic drugs (1). We present a psoriasis patient developed MEDFs following sequential treatments with secukinumab, guselkumab, and adalimumab. Although anti-interleukin(IL)-17, IL-23p19 and anti-TNF-α therapy is rarely implicated with MEDFs (3), causality assessment classified as drug reaction in this case (4), considering the appearance of DF in response to the intensity and time of biological treatment.

## Case report

**Figure 1:**
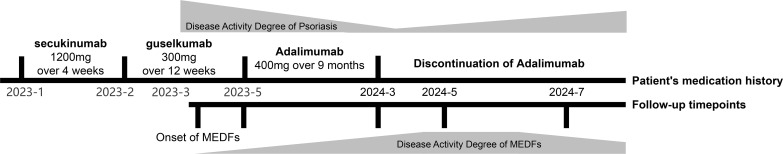
Timeline of clinical progression and therapeutic interventions.

An otherwise healthy male in his twenties, with a 12-year history of refractory psoriasis, developed multiple cutaneous tumors following sequential biologic therapies: secukinumab (cumulative dose of 1200mg over 4 weeks), guselkumab (cumulative dose of 300mg over 12 weeks), and adalimumab (cumulative dose of 400mg over 9 months). The patient showed poor response to secukinumab, and significant improvement of psoriasis was observed only upon initiation of the second course of guselkumab treatment, however, widespread asymptomatic skin lesions appeared (occurring two weeks post-second guselkumab injection), prompting a switch to adalimumab. Despite the further response to adalimumab in managing the patient’s psoriasis, therapy was terminated during the tenth cycle owing to the deterioration of cutaneous neoplasms (**Figure 1**). The patient reports no known significant family history of chronic diseases or genetic disorders in first-degree relatives. He denies current or recent use of any medications other than biological agents, oral desloratadine, and topical fluticasone cream (specifically excluding immunosuppressants, aspirin, ibuprofen, herbal remedies, or supplements). Additionally, he has no history of hypertension, diabetes mellitus, coronary artery disease, asthma, chronic obstructive pulmonary disease, thyroid disorders, or malignancy. As a university student, he denies tobacco use, alcohol consumption, and significant psychosocial stressors.

**Figure 2:**
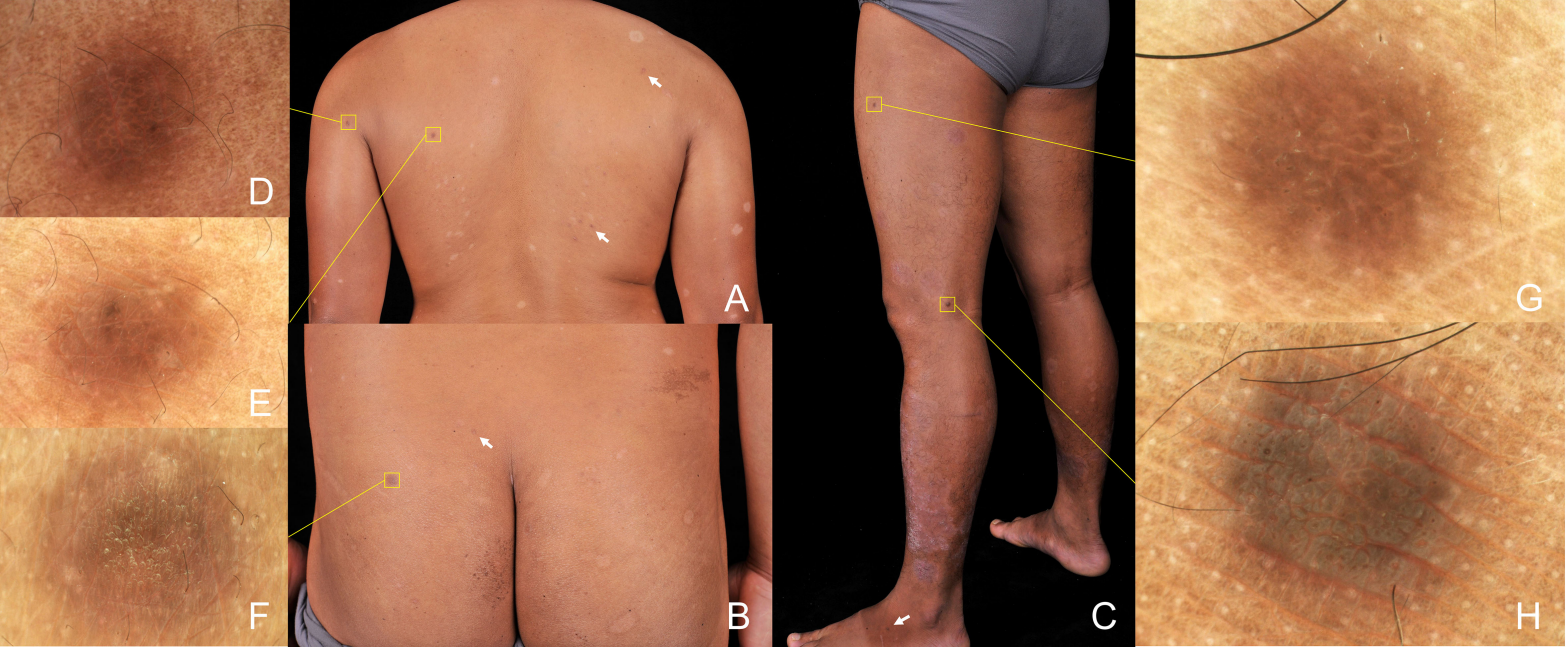
Physical examination shown scaly erythematous plaques on lower limbs, as well as multiple well-demarcated, firm, brown papules and nodules randomly distributed in extremities and trunk (box and arrow) **(A-C)**. Dermoscopic evaluation of all lesions demonstrated features of dermatofibromas, including central white striate scar-like structures surrounded by a delicate pigmented network, irregular crypts, and pseudofollicular openings (polarizer, original magnification, ×20) **(D–H)**.

**Figure 3:**
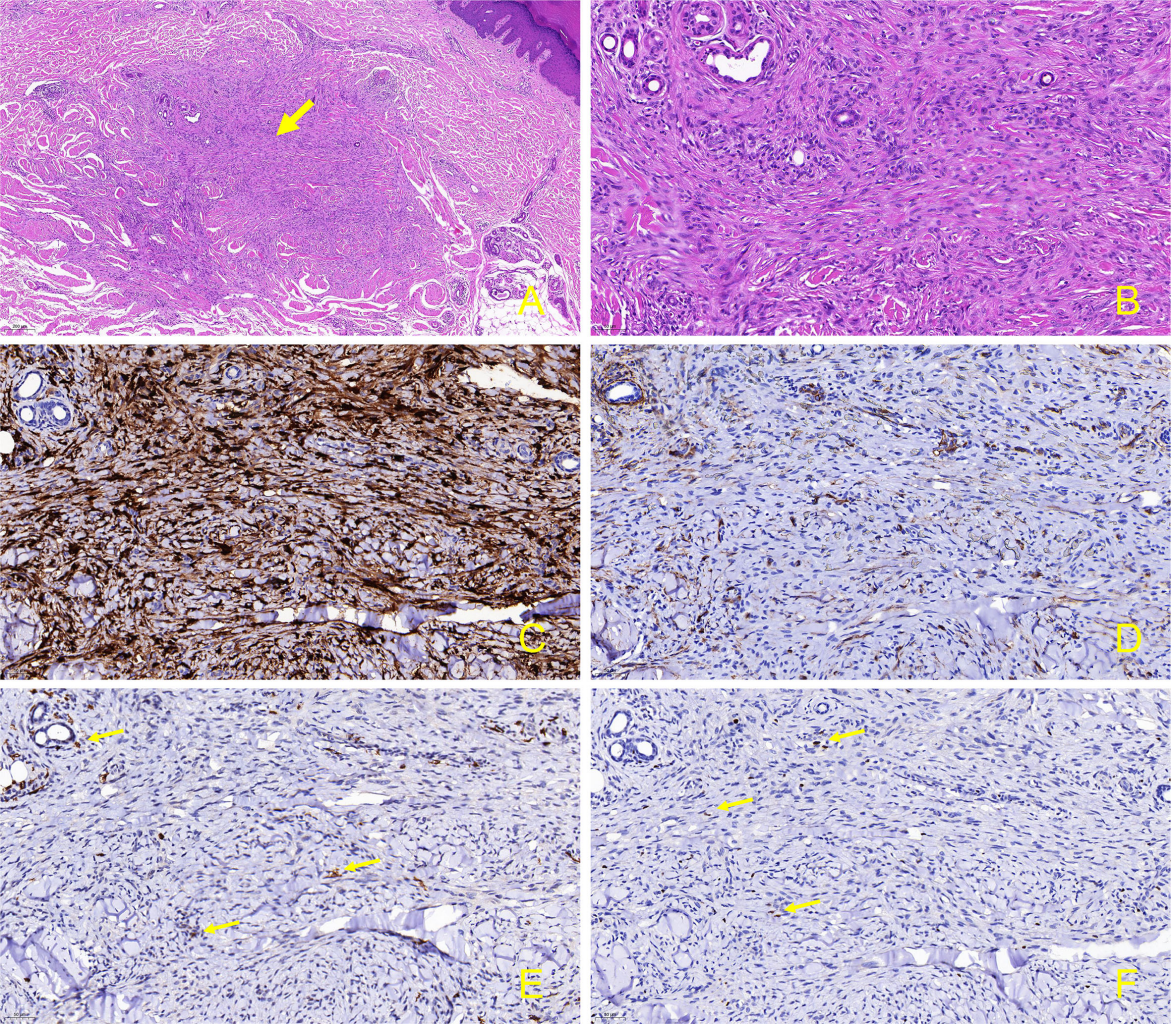
Skin biopsy and immunohistochemical stain from the lesion on the back. **(A)** Proliferation of fibrohistiocytic spindle-shaped cells in a whorled and fascicular pattern, some of which are surrounding thickened collagen fibers in the reticular dermis (hematoxylin-eosin, original magnification, ×50); **(B)** Spindle-shaped cells are entrapped within thickened bundles of collagen. (hematoxylin-eosin, original magnification, ×200); **(C)** Factor XIIIa is positive in the spindle-shaped tumor cells. (immunostains, original magnification, ×200); **(D)** CD34 is negative in the tumor cells (immunostains, original magnification, ×200); **(E)** CD117 exhibits positivity in the membrane and cytoplasm of mast cells infiltrating tumor tissues. (immunostains, original magnification, ×200, arrow); **(F)** Within the tumor tissue, occasional lymphocytes surrounding blood vessels exhibit positivity for GATA3. (immunostains, original magnification, ×200, arrow).

Physical examination revealed scaly erythematous plaques on his lower limbs, as well as multiple well-demarcated, firm, reddish-brown papules and nodules on extremities and trunk predominantly appearing in areas previously affected by psoriatic dermatitis (**Figures 2A–C**). Dermoscopic evaluation of all lesions demonstrated a central white scar-like area or diffused hypopigmentation surrounded by a reddish-brown pigment network, with seborrheic keratosis-like patterns (irregular crypts and pseudofollicular openings) discernible in some lesions (**Figures 2D–H**). Histopathological findings of a nodule from the back revealed proliferation of fibrohistiocytic spindle cells arranged in whorled and fascicular patterns, encircling thickened collagen bundles within the reticular dermis (**Figure 3A**), and spindle-shaped cells entrapped within thickened bundles of collagen (**Figure 3B**). Immunohistochemistry (IHC) demonstrates tumor cell positivity for Factor XIIIa (RRID: AB_2881706) (**Figure 3C**) but negativity for CD34 (RRID: AB_10733337) (**Figure 3D**), with infiltrating mast cells exhibiting membranocytoplasmic CD117 (RRID: AB_2249558) immunoreactivity (**Figure 3E**) and scattered intratumoral lymphocytes showing occasional nuclear GATA3 (RRID: AB_2881774) expression (**Figure 3F**).

**Figure 4:**
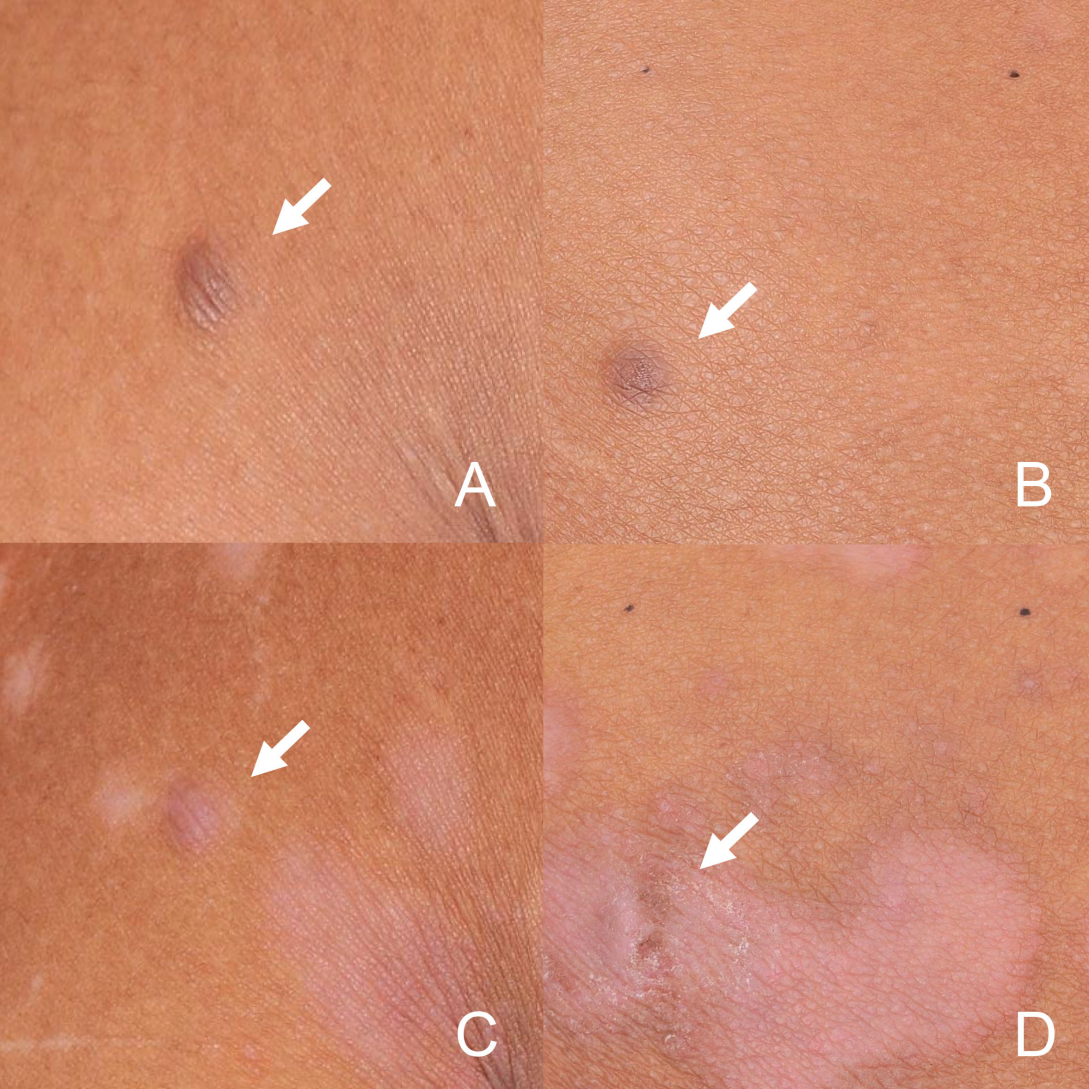
Dermatofibromas in the back **(A)** and buttocks **(B)** at baseline. During the five-month discontinuation of adalimumab period, dermatofibromas in back **(C)** and buttocks **(D)** slightly shrank. (arrow).

Given the clinical, dermoscopic and histopathological features, the current patient was diagnosed as MEDFs. The Naranjo algorithm (total score of 7) and WHO-UMC scale classified the causality as a “probable” adverse drug reaction (ADR) (4) in this case, considering the appearance of DF in response to the intensity and time of biological treatment (**Supplementary Materials 1**). Subsequently, adalimumab was discontinued. During the five-month follow-up period, dermatofibromas slightly shrank, with no new lesions being observed, however, psoriatic lesions recurred in trunk and limbs (**Figures 4A–D**). As the patient was asymptomatic, no specific treatment was administered, but regular monitoring was advised.

## Discussion

This case report and literature review collectively demonstrate an emerging association between biologic therapies and MEDFs, particularly in patients with psoriasis. The development of MEDFs during biologic therapy for psoriasis, including anti-IL-23 agents (ustekinumab, n = 2) (5, 6), anti-TNF-α agents (etanercept and adalimumab, n = 1) (3), and anti-CD11a therapy (efalizumab, n = 1) (7), has been reported. Consistent with prior reports, MEDFs emerged during biologic treatment, presenting shared features of asymptomatic reddish-brown papules/nodules on trunk/extremities and CD34^−^/FXIIIa^+^ histology. The rapid onset following guselkumab (2 weeks) initiation differs from1–8 month latency reported in other cases. While all cases demonstrated lesion cessation upon therapeutic discontinuation, this patient uniquely received sequential IL-17A/IL-23/TNFα inhibition with MEDFs progression despite therapy switch, implying cumulative immune dysregulation.

Clinically, DF typically manifests as a firm, solitary, hyperpigmented nodule preferentially affecting the lower limbs. Dermoscopic features of DF vary, but typically, central white striate or patchy scar-like structures surrounded by a delicate pigmented network can be observed. Histopathologically, DF present as well-circumscribed, nonencapsulated dermal nodules consisting of spindle-shaped fibrohistiocytic cells intermingled with homogenized eosinophilic collagen bundles, accompanied by overlying epidermis acanthosis and basal layer hyperpigmentation (1). Differential diagnosis of MEDFs mainly encompasses dermatofibrosarcoma protuberans (DFSP), cutaneous piloleiomyoma, and Kaposi’s sarcoma (KS). DFSP is a soft tissue sarcoma, presenting as an asymptomatic, flesh-colored, indurated plaque that gradually develops into a multinodular lesion on the trunk and proximal extremities. Piloleiomyoma is a benign tumor originating from the arrector pili muscle, typically presenting as solitary or multiple painful nodules. In tumor cells of the lesion, the factor XIIIa positivity confirms dermal dendrocyte differentiation, the neoplastic component central to dermatofibroma formation, while CD34 negativity excludes vascular proliferation and aids in distinguishing MEDFs from DFSP. KS is an angioproliferative tumor induced by human herpesvirus 8, predominantly affecting HIV-infected individuals. It presents as cutaneous lesions varying from macules, plaques, nodules to exophytic growths, with or without internal involvement. Immunocompromised patients may clinically confuse MEDFs with other papular lesions such as leukemic skin infiltration, bacillary angiomatosis, among others (8). Accurate diagnosis relies on histological and immunohistochemical examinations. In this patient, signs of malignancy, including rapid growth, ulceration, or systemic symptoms, were absent. Additionally, piloleiomyoma was ruled out by the absence of paroxysmal pain attacks. Histopathology and IHC (FXIIIa^+^/CD34⁻) conclusively excluded DFSP, KS, and piloleiomyoma. Given the clinical features, the current patient fulfilled the diagnosis of MEDFs.

MEDFs are well-established as an abortive immuno-response mediated by antigen-presenting cells (APCs), characterized by fibroblast proliferation and collagen deposition (9). In this case, definitive attribution of MEDFs could not be established beyond clinical assessment. Based on established pathways reported in existing literature, the following speculative pathophysiological hypothesis are proposed. Theoretically, blockage of IL-17, IL-23p19, or TNF could upregulate Foxp3^+^Treg cells (Tregs) (10–12), increasing anti-inflammatory cytokine levels, which may contributing to persistent activation of APC due to failure to eliminate cutaneous antigens or cytokines, triggering the onset of MEDFs (13). As supporting in earlier work, the sequential use of biologic agents targeting Th1/Th17-driven inflammatory cascade promote a shift in Th1/Th2 balance towards a Th2-skewing immune microenvironment (14, 15). Prior research demonstrates that Th2 cytokines (IL-4, IL-13) promote fibrosis via STAT6-dependent pathways in chronic inflammatory diseases, while concomitant TGF-β signaling amplifies extracellular matrix production and myofibroblast differentiation (16), which potentially contributing to MEDFs development. Moreover, certain Th2 mediators have been implicated in stimulating the mitogenic and synthetic activity of fibroblasts. These include mast cells, eosinophils, IL-5, factor XIIIa, and histamine, which are considered integral to the pathogenesis of MEDFs (9). In immunohistochemical staining of the patient’s lesion, the expression of CD117 in mast cells suggests their putative role in stromal remodeling. Infiltration of scattered GATA3^+^ lymphocytes implylocalized Th2 polarization, consistent with the cytokine microenvironment (e.g., IL-4/IL-13) implicated in fibroblast recruitment and collagen deposition (**Supplementary Materials 2**). However, there is currently no direct evidence linking the development of MEDFs to a Th2 immune shift. The exact triggering factors for MEDFs in the present case remain unclear. The cumulative effects of several biologics, profound Th2-driven effects mediated by targeting the upstream of the Th1/Th17 axis, TNF blockage, Treg inhibition, et cetera may be involved.

There are several limitations in this case. To definitively identify the implicated biologic agent among the three administered (adalimumab, ustekinumab, secukinumab) for MEDFs was challenging due to concurrent exposure before symptom onset. While ADR causality assessment tools (the Naranjo and WHO-UMC scales) suggested probable biologic involvement, their application in sequential multi-drug exposure remain constrained, primarily by their reliance on temporality assessments and inability to differentiate synergistic or sequential interactions. Furthermore, the inherent limitations in generalizing single-case findings necessitate larger cohort studies orthe exploration of pharmacovigilance databases to validate the pathogenicity and incidence of MEDFs associated with these biologics. Additionally, future investigations using cytokine/transcript profiling in similar cases are warranted. The emergence of novel side effects from biological therapies, not identified during clinical trials, should raise concern.

## Take-away

1. MEDFs are diagnosed when 5–8 dermatofibromas develop within 4 months, with distinct clinical, dermoscopic, and histopathological features for accurate diagnosis.

2. MEDFs indicate an underlying immune alteration, often associated with immune-mediated disorders or medications.

3. Long-term TNF blockade and Th1/Th17 axis suppression may contribute to MEDFs’ pathogenesis, emphasizing the need for vigilance in monitoring adverse drug reactions.

## Data Availability

The original contributions presented in the study are included in the article/**Supplementary Material**. Further inquiries can be directed to the corresponding author.
