# Supplementary material for: Multiple eruptive dermatofibromas occurred after receiving sequential treatment with secukinumab, guselkumab, and adalimumab: case report
Source: Front Immunol. 2025 Nov 7;16:1611831. doi: 10.3389/fimmu.2025.1611831 (PMC12635618; doi:10.3389/fimmu.2025.1611831)
Supplement: Supplementary file 2 [file Table1.docx]

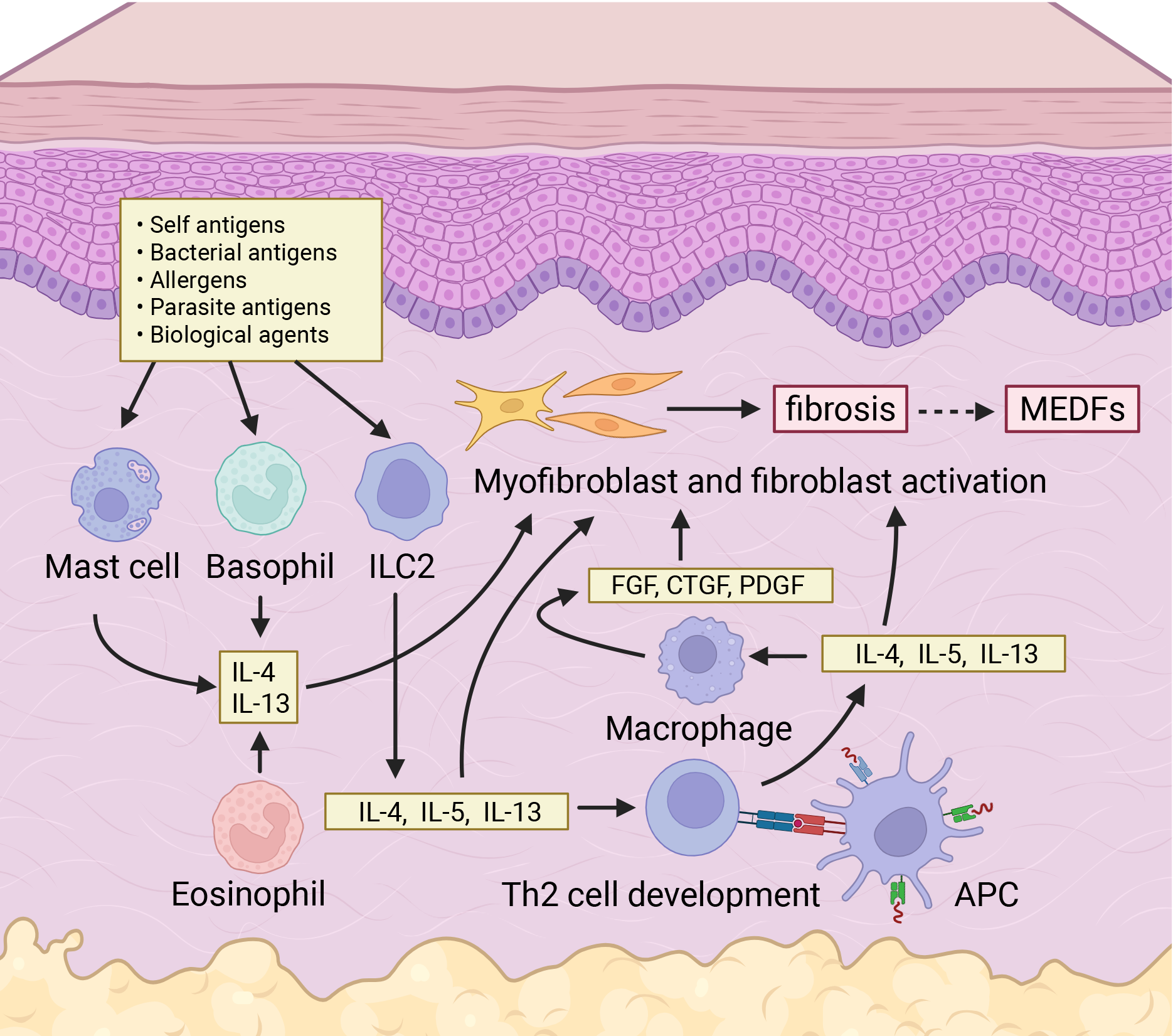


**Supplementary File 2 Mechanistic hypothesis of type 2 immune responses in MEDFs.**

Under the influence of triggering factors, innate cells including basophils, mast cells, and group 2 innate lymphoid cells (ILC2s) produce type 2 cytokines (IL‑4, IL‑5, and IL‑13). These cytokines drive naive CD4⁺ T cell differentiation into T helper 2 (Th2) cells and enhance Th2 effect. Concurrently, antigens are presented to T cells by antigen-presenting cells (APCs). IL‑4 and IL‑13 secreted from basophils, mast cells, eosinophils, and ILC2s further directly activate myofibroblasts and promote fibrosis. Collectively, extracellular matrix production and myofibroblast differentiation potentially contribute to MEDF development. In schematic representations, solid lines denote established pathways, while dashed lines indicate proposed pathways. (Adapted from Gieseck RL III, Wilson MS, Wynn TA. Type 2 immunity in tissue repair and fibrosis. Nat Rev Immunol. 2018;18(1):65, doi:10.1038/nri.2017.90. The figure was generated using BioRender.)
